# Pain characteristics of adolescent spinal pain

**DOI:** 10.1186/s12887-015-0344-5

**Published:** 2015-04-17

**Authors:** Brigitte Wirth, B Kim Humphreys

**Affiliations:** Institute of Human Movement Sciences and Sport, ETH Zurich, Wolfgang Pauli Str. 27, 8093 Zurich, Switzerland; Chiropractic Department, University of Zurich and University Hospital Balgrist, Forchstr. 340, 8008 Zurich, Switzerland

**Keywords:** Adolescence, Pain characteristics, Spinal pain

## Abstract

**Background:**

Although adolescent spinal pain increases the risk for chronic back pain in adulthood, most adolescents can be regarded as healthy. The aim of the present study was to provide data on localization, intensity and frequency of adolescent spinal pain and to investigate which physical and psycho-social parameters predict these pain characteristics.

**Method:**

On the occasion of Spine Day, an annual event where children and adolescents are examined by chiropractors on a voluntary basis for back problems, 412 adolescents (10 to 16 years) were tested (by questionnaire and physical examination). Pain characteristics (localization, intensity, and frequency) were identified and evaluated using descriptive statistics. Regression analyses were performed to investigate possible influencing psycho-social and physical influence factors.

**Results:**

Adolescents who suffered from pain in more than one spinal area reported higher pain intensity and frequency than those with pain in only one spinal area. Sleep disorders were a significant predictor for pain in more than one spinal area (p < 0.01) as well as a trend for frequent pain (p = 0.06). Adolescents with frequent pain showed impaired balance on one leg standing with closed eyes (p = 0.02).

**Conclusions:**

Studies on adolescent spinal pain should report data on pain frequency, intensity and localization. Adolescents who present with pain in more than one spinal area or report frequent pain should be followed carefully. Reduced balance with visual deprivation might be a physical indicator of a serious back problem.

**Electronic supplementary material:**

The online version of this article (doi:10.1186/s12887-015-0344-5) contains supplementary material, which is available to authorized users.

## Background

Back pain starts early in life and its prevalence increases with age, accelerating in the early teens around the age of 12 to 15 [1-3] and reaching adult prevalence by the age of 18 [1,4]. As for low back pain (LBP), an eight-year follow up from adolescence to adulthood showed a fourfold increase in the risk of adolescent LBP becoming LBP in adult life. The longer the duration of LBP in adolescence, the higher was the risk for persistent LBP in adulthood [5]. Thus, it was postulated that the focus of research, prevention and treatment in this area should be changed from the adult to the young population [5].

Nevertheless, fostering fear-avoidance beliefs by medicalization of the problem should be avoided [6]. It has been reported that the vast majority of adolescents with LBP should be considered as healthy, because LBP had little effect on health-related quality of life [7]. A recent study in this field identified four co-morbidity clusters in adolescents with LBP at the age of 17 years [8]: About 80% of the adolescents could be assigned to the ‘healthy individuals cluster’. These subjects had low probability of being diagnosed with LBP or any other medical condition and the experience of LBP could be considered as relatively benign. About 10% of the adolescents were in the ‘spinal pain cluster’, indicating high probability of being diagnosed with both LBP and headaches, in the absence of other co-morbidities such as sleep disorders or psychological problems, which are common symptoms of widespread pain disorders. In contrast, the subjects in the ‘LBP and depression/anxiety disorders cluster’ (7%) and those in the ‘LBP and behavioral/attention disorders cluster’ (4%) exhibited an increased probability of having diagnosed sleep disorders and headaches along with the corresponding psychological problems. The findings in the latter two clusters were linked to a possible dysregulation of the hypothalamic-pituitary-adrenal axis [8].

It has been proposed that in order to optimize intervention strategies, researchers and clinicians in the field of adolescent spinal pain should discriminate between children with negligible, benign back pain and those with a high risk for developing chronic spinal pain [9]. Consequently in addition to prevalence data, more concise characterization of adolescent spinal pain such as frequency, pain intensity and consequences/resulting disability is needed [9]. However, most studies, particularly those investigating physical risk factors for adolescent spinal pain, have thus far been restricted to reporting prevalence measures of back pain without considering its severity. This has led to the conclusion that psychosocial factors might be more important than mechanical factors for LBP in young populations [10].

Therefore, the aim of the present study was 1) to describe intensity, frequency and localization of spinal pain and resulting disability in adolescents between 10 and 16 years and 2) to investigate which physical and psycho-social parameters predict these pain characteristics.

## Methods

### Participants

Since 2006 in November, on the occasion of the WHO’s International Spine Day, the Swiss Chiropractic Association (ChiroSuisse) has organized an action day where Swiss school children and adolescents are examined on a voluntary and free of charge basis for spinal problems. The event is advertised in print and electronic media and by flyers that are distributed in chiropractic practices. Ethics approval was obtained from all the cantons of Switzerland that required approval for this type of study (BS/BL, FR, LU, SG, VD and the Ethics committee of ETH Zurich). The subjects of the current study were all participants of Spine Day in the age group of interest (between 10 and 16 years) whose parents (or the legal representatives) gave written consent that the anonymized data of their children could be included in this study.

Eighty-four chiropractors volunteered for Spine Day 2013. In total, 860 children and adolescents were examined, of whom 412 were in the age group of interest (10y: N = 80; 11y: N = 72; 12y: N = 73; 13y: N = 67; 14y: N = 63; 15y: N = 42; 16y: N = 15). Consequently, 198 boys and 214 girls (mean age = 12.4 ± standard deviation 1.8 years, mean height = 1.56 ± 0.12 m, mean BMI = 18.8 ± 3.2 kg/m^2^) were included in the data analysis.

### Procedure

The participants and their parents or representatives were asked to complete a questionnaire, which is shown in an additional file (Additional file 1). Apart from demographic information (sex, age), the questionnaire characterized spinal pain by its prevalence, frequency, intensity and consequences, as recommended in the literature [9]. The *lifetime prevalence* and the *one-month period prevalence* of spinal pain were filled out separately by the affected spinal region(s) (low back pain (LBP), neck pain, middle back pain and pain in more than one spinal area) [1,2] as illustrated by a manikin. The one-month period was recommended as a reliable recall period [1] and has been applied in various studies [7,11]. The *frequency* of spinal pain in the last month was classified as ‘once’, ‘seldom (few days)’, ‘often (several days)’, ‘daily’. The *intensity* of spinal pain was quantified using a visual analog scale (VAS) ranging from 0 (no pain) to 10 (intolerable pain). The *consequences* of spinal pain were assessed as a reduction of leisure activities, school absence, seeing a doctor/chiropractor or taking medication [9]. As for psycho-social and lifestyle factors, the *spinal pain history of parents* (mother yes/no; father yes/no), the duration of *TV/computer activities* in leisure time per week and the *smoking habits* of the participant and his/her parents were asked for as all these factors have been associated with an increased risk for spinal pain [3,12]. As very few participants smoked themselves, only the influence of parental smoking habits was analyzed, which has been reported to be a possible risk factor for spinal pain [13,14]. Lastly, the participants were asked for *other somatic symptoms* (headache, abdominal pain apart from period pains, headache and abdominal pain) and *sleep disorders* in the past month. A high number of symptomatic symptoms was reported to be a risk for developing low back pain [10,15], while sleep disturbance might point to an increased stress level [8].

The physical investigation was similar to Spine Day 2012 [14]. The chiropractors first measured body height and weight. *Trunk symmetry* was assessed by the Adam’s forward bend test. This test is the most widely used test in school scoliosis screening [16] and is rated positive, if a subjects presents with a rib hump while bending forward from a standing position. Trunk asymmetry and the diagnosis of scoliosis were reported to increase the risk for LBP [14,17,18], but the significance of these parameters requires further investigation [18,19]. *Coordination* was assessed by asking the participants to stand 10 seconds on each leg, once with eyes open and once with eyes closed [20]. In adults with LBP, several studies have reported increased center of pressure (COP) sway [21], particularly with closed eyes [22]. Thus, only the single leg stance with closed eyes was included in the further analyses. *Mobility* was tested by measuring finger to floor distance (FFD) when bending forward from a standing position [23] with knee, arms and fingers extended, which is considered to be a measure for combined spine and pelvic mobility [23]. It was not differentiated whether a participant touched the floor with the finger tips or with flat hands. The chiropractors also visually assessed static deficits in spinal posture and in the knees and feet, investigated range of spinal mobility and tested whether palpation of the vertebrae was painful, but these data were not included in the analyses.

### Data analysis and statistics

Localization, intensity and frequency of spinal pain and resulting disability were described using descriptive statistics. The 95% confidence intervals (CI) were calculated with the Clopper Pearson method. For the definition of mild, moderate and severe levels of pain, cut-off points of 35:60 were chosen [24]. For the investigation of the relation between pain intensity and pain localization and frequency, one-way ANOVA with post hoc Tukey HSD tests were conducted. For investigating the impact of spinal pain on daily life, pain intensity of those adolescents who undertook some actions due to spinal pain was compared using the unpaired *t*-test to those who did not change their daily life. Frequency and consequences of spinal pain were compared in the different spinal areas using a *χ*^2^-test. Furthermore, a logistic regression (forced entry/enter method) was performed to examine if pain intensity, localization or frequency had an impact of whether spinal pain affected an adolescent’s daily life (coding 0 = no consequences, 1 = consequences).

For the investigation of possible influencing factors on the variables pain intensity, frequency and localization, the following 13 factors were included in the regression analyses: 1) continuous parameters: age, time spent in front of TV/computer per week, BMI, FFD and 2) binary variables (coding 0/1): gender (male/female), Adams forward bending test (absence/presence of rib hump), single leg stance for 10 seconds with closed eyes (possible on both legs/not possible on one or both legs), parental spinal pain (no/yes; no differentiation whether mother and/or father has pain), smoking parents (no/yes; no differentiation whether mother and/or father smokes), sleep problems (no/yes), headache (no/yes), abdominal pain (no/yes), headache in combination with abdominal pain (no/yes). In addition to the multivariate model, also the values for the unadjusted/crude odds ratio were calculated. A linear regression analysis was conducted to examine the influencing factors on pain intensity. (Multinomial) logistic regression analyses were performed to investigate for possible influence of the factors on pain frequency and pain localization and on the combination of these variables. As there were only 8 adolescents with mild, but frequent back pain, this group was not included in the corresponding multinomial logistic regression analysis. For the analyses on pain characteristics, data sets with missing values were excluded from the corresponding analyses (available case-analysis). For the regression analyses, only complete data sets were included (complete case analysis) and the models were checked for multi-collinearity. All analyses were conducted using SPSS 20. The significance level was set at p < 0.05.

## Results

### Characteristics of spinal pain

Of the 412 adolescents, 183 (44.4%; 95% CI: 39.6, 49.4) indicated that they had suffered from back pain within the last month. At the age of 10, 30 of 80 participants (30.0%; 95% CI: 20.3, 41.3; 3 missing data) reported back pain in the last month. At the age of 11, 32 of 72 participants (44.4%; 95% CI: 32.7, 56.6; 2 missing data) and at the age of 12, 25 of 73 adolescents (34.3%; 95% CI: 23.5, 46.3; 1 missing data) complained about back pain in the last month. At the age of 13, 35 of 67 participants (52.2%; 95% CI: 39.7, 64.6; 1 missing data) and at the age of 14, 38 of 63 adolescents (60.3%; 95% CI: 47.2, 72.4; no missing data) reported back pain in the last month. Lastly, at the age of 15, 23 of 42 participants (54.8%; 95% CI: 38.7, 70.2; 3 missing data) and at the age of 16, 6 of 15 adolescents (40.0%; 95% CI: 16.3, 67.7; 3 missing data) suffered from back pain in the last month. As for localization, 45 (10.9%; 95% CI: 8.1, 14.3) subjects experienced neck pain, 35 (8.5%; 95% CI: 6.0, 11.6) middle back pain, 51 (12.4%; 95% CI: 9.4, 16.0) LBP, while 52 (12.6%; 95% CI: 9.6, 16.2) adolescents suffered from pain in more than one spinal area (13 missing data). Both, non-recurrent (N = 26/6.3%; 95% CI: 4.2, 9.1) and daily pain (N = 12/2.9%; 95% CI: 1.5, 5.0) were rare. The majority of adolescents reported experiencing back pain on a few days/seldom (N = 89/21.6%; 95% CI: 17.7, 25.9) or on several days/often (N = 60/14.6%; 95% CI: 11.3, 18.4) (9 missing data). Pain intensity ranged from 1.0 to 9.0 points on the VAS scale, resulting in a mean of 4.5 (±SD 1.7) points (11 missing data). According to the definition by Hirschfeld and Zernikow [24], of the 183 adolescents with spinal pain, 26 (14.2%; 95% CI: 9.5, 20.1) suffered from severe pain (VAS score > 6.0), while 102 subjects (55.7%; 95% CI: 48.2, 63.1) reported their pain as moderate (VAS score between >3.5 and ≤ 6.0) [24]. In the majority of subjects, spinal pain did not result in any consequences. Nevertheless, 55 adolescents indicated to have changed something in their daily life due to their back pain: 12 took pills, 11 reduced their (sport) activities, 8 went to see a doctor, 7 chose more than one answer and 15 indicated other actions, such as being massaged by the parents.

**Figure 1:**
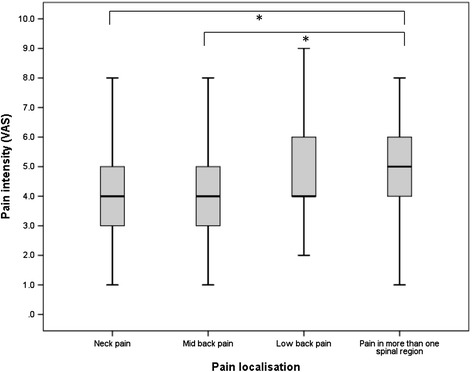
Pain intensity in the spinal regions. Pain in more than one spinal region was significantly higher than neck pain or mid back pain.

**Figure 2:**
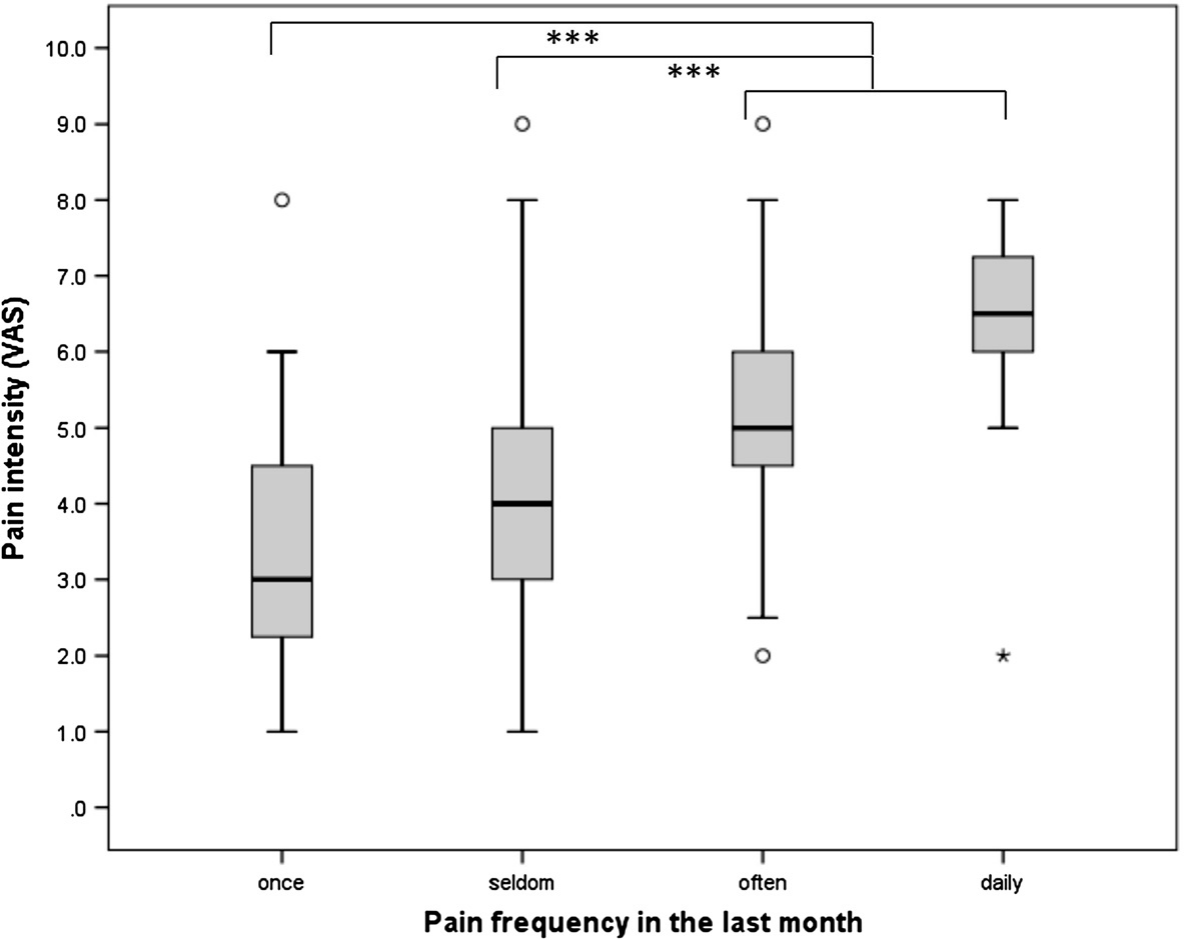
Pain intensity and pain frequency. Daily and frequent pain was of significantly higher intensity than non-recurring or occasional pain.

**Figure 3:**
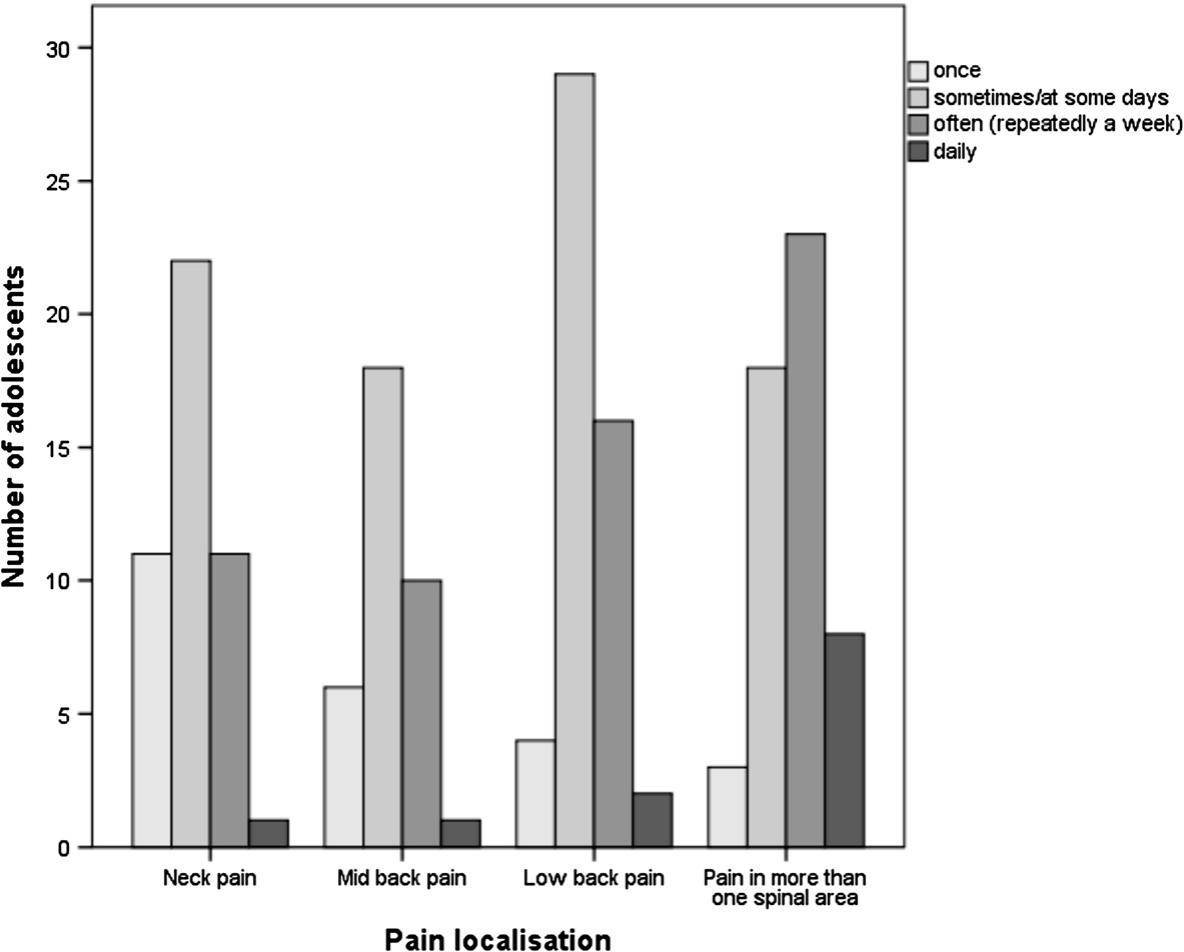
Pain frequency in the spinal regions. Frequent and daily pain was very common when pain was reported in more than one spinal region.

The intensity of neck (4.0 ± 1.7 VAS points) and mid back pain (4.0 ± 1.5 VAS points) was the lowest on average, followed by LBP (4.7 ± 1.8 VAS points) and pain in multiple spinal areas (5.1 ± 1.7 VAS points) (Figure 1). The one-way ANOVA revealed a significant difference in pain intensity for the different regions (F_(3,177)_ = 4.68, p = 0.004). Post hoc comparisons using the Tukey HSD test indicated that neck and mid back pain intensity significantly differed from the intensity of pain in more than one spinal area (p = 0.010 and p = 0.024, respectively). LBP intensity did not significantly differ from any of the other spinal regions. Pain intensity became higher and was statistically significant (F_(3,181)_ = 21.14, p < 0.001) when pain frequency was also increased (Figure 2). Non-recurrent pain was experienced on average as mild (3.4 ± 1.7 VAS points), occasional (seldom/at few days) (3.9 ± 1.4 VAS points) and frequent (often/at several days) pain (5.4 ± 1.5 VAS points) as moderate, while daily pain was severe (6.3 ± 1.6 VAS points). The intensity of non-recurrent and occasional pain thereby significantly differed from that of frequent and daily pain (p < 0.001). Adolescents who undertook some action due to their spinal pain indicated significantly higher pain intensity (5.1 ± 1.8 VAS points) than those whose spinal pain did not affect daily life (4.2 ± 1.6 VAS points) (p = 0.002). The adolescents with pain in more than one spinal area indicated a higher proportion of frequent (44.2%; 95% CI: 30.5, 58.7) and daily pain (15.4%; 95% CI: 6.9, 28.1) than those with pain in the neck (frequent: 24.4%, 95% CI: 12.9, 39.5; daily: 2.2%, 95% CI: 0.1, 11.8), in the mid back (frequent: 28.6%, 95% CI: 14.6, 46.3; daily: 2.9%, 95% CI: 0.1, 14.9), or in the lower back (frequent: 31.4%, 95% CI: 19.1, 45.9; daily: 3.9%, 95% CI: 0.5, 13.5) (*χ*^2^ = 22.94, p = 0.006) (Figure 3). Consequently, pain in more than one spinal area led to more changes in daily life (neck pain: 24.4%, 95% CI: 12.9, 39.5; mid back pain: 22.9%, 95% CI: 10.4, 40.1; LBP: 27.5%, 95% CI: 15.9, 41.8; pain in more than one spinal area: 41.2%, 95% CI: 27.6, 55.8). However the difference was not significant (*χ*^2^ = 4.72, p = 0.193). In terms of the impact on daily life, pain intensity was the only predictor of whether the back pain experienced resulted in any disability or consequence. One point on the VAS scale thereby increased the risk for undertaking actions due to spinal pain by the factor 1.4 (p = 0.007, Nagelkerke R^2^ = 0.13).

### Influencing factors on pain intensity, frequency and localization

The absolute values of all possible predictors that were investigated are shown in Table 1. Multi-collinearity was detected in any of the regression models (tolerance values between 0.77 and 0.97; variance inflation factor values between 1.03 and 1.30). Age, the presence of headache in combination with abdominal pain and the presence of headache only were found to be the significant predictors for pain intensity (R^2^ = 0.15) (Table 2). Adolescents with non-recurring or occasional pain differed from those without spinal pain within the last month in terms of age and the presence of parental back pain. Adolescents with frequent or daily pain were older than those without spinal pain in the last month, complaint also about abdominal pain and headache or headache only and showed significantly less ability to stand 10 seconds with closed eyes on one leg (Nagelkerke R^2^ = 0.21) (Table 3). Lastly, age, TV/computer activities, headache in combination with abdominal pain and sleep disorders were the parameters that additionally distinguished adolescents with pain in more than one spinal area from the pain-free adolescents (Nagelkerke R^2^ = 0.28) (Table 4).

**Table 1:** **Absolute values of the predictors for the adolescents without spinal pain, for those with pain in one spinal area and for those with pain in more than one spinal area**

	**No spinal pain (N = 216)**	**Pain in one spinal area (N = 131)**	**Pain in more than one spinal area (N = 52)**
Age (mean/SD)	12.0 ± 1.7	12.6 ± 1.8	12.9 ± 1.6
Gender (male/female)	116/100	60/71	19/33
BMI (mean/SD)	18.4 ± 3.4	19.1 ± 2.9	19.1 ± 2.5
Finger Floor Distance (mean/SD)	10.0 ± 9.8	9.5 ± 10.9	8.8 ± 9.7
Adams sign (Asymmetry/Symmetry)	29/176	27/101	11/40
Single leg stance with closed eyes (reduced/ok on both legs)	41/170	25/103	10/40
TV/computer activities (mean/SD)	7.1 ± 5.9	8.2 ± 7.4	9.3 ± 6.3
Parental back pain (yes/no)	157/26	103/7	39/5
Parental smoking (yes/no)	48/156	34/88	12/39
Sleep disorders (yes/no)	40/173	36/94	23/29
Headache (yes/no)	39/173	34/96	20/32
Abdominal pain (yes/no)	23/189	13/117	4/48
Headache and abdominal pain (yes/no)	14/198	22/108	11/41

BMI = body mass index.

**Table 2:** **Predictors for pain intensity (linear regression analysis)**

	**B (SE)**	**Standardized Coefficient**	**p**
Age	0.32 (0.09)	0.23	**<0.001**
Gender	0.32 (0.29)	0.07	0.262
BMI	0.01 (0.05)	0.01	0.913
Finger Floor Distance	−0.02 (0.01)	−0.07	0.217
Adams sign	−0.13 (0.37)	−0.02	0.721
Single leg stance with closed eyes	0.58 (0.34)	0.10	0.092
TV/computer activities	0.02 (0.02)	0.06	0.315
Parental back pain	0.29 (0.42)	0.04	0.500
Parental smoking	0.43 (0.32)	0.07	0.184
Sleep disorders	0.46 (0.33)	0.08	0.165
Headache	0.86 (0.34)	0.15	**0.011**
Abdominal pain	0.32 (0.48)	0.04	0.503
Headache and abdominal pain	1.49 (0.44)	0.20	**0.001**

BMI = body mass index.

Numbers in bold indicate statistical significance.

**Table 3:** **Predictors for pain frequency (multinomial logistic regression analysis)**

	**Non-recurring or occasional pain (N = 90) versus no pain (N = 160)**				**Frequent or daily pain (N = 52) versus no pain (N = 160)**			
	**B (SE)**	**OR:**	**95% CI**	**p**_**adj**_	**B (SE)**	**OR:**	**95% CI**	**p**_**adj**_
		**Exp B**_**adj**_ **(Exp B**_**unadj**_ **)**	**Exp B**_**adj**_			**Exp B**_**adj**_ **(Exp B**_**unadj**_ **)**	**Exp B**_**adj**_	
Age	0.26 (0.09)	1.30 (1.24)	1.09-1.56	**0.004**	0.28 (0.11)	1.33 (1.26)	1.07-1.65	**0.011**
Gender	−0.01 (0.30)	0.99 (0.82)	0.55-1.76	0.963	−0.60 (0.37)	0.55 (0.41)	0.27-1.14	0.106
BMI	0.05 (0.05)	1.05 (1.08)	0.96-1.15	0.326	−0.01 (0.06)	0.99 (1.07)	0.88-1.11	0.827
Finger Floor Distance	−0.02 (0.02)	0.98 (0.99)	0.96-1.01	0.275	−0.01 (0.02)	0.99 (1.00)	0.96-1.03	0.760
Adams sign	−0.07 (0.38)	0.93 (0.72)	0.45-1.95	0.855	−0.09 (0.45)	0.92 (0.51)	0.38-2.20	0.845
Single leg stance with closed eyes	0.12 (0.38)	1.12 (1.27)	0.53-2.37	0.762	−0.92 (0.40)	0.40 (0.64)	0.18-0.88	**0.023**
TV/computer activities	0.03 (0.02)	1.03 (1.04)	0.99-1.07	0.177	0.02 (0.03)	1.02 (1.04)	0.97-1.08	0.365
Parental back pain	−1.16 (0.53)	0.31 (0.33)	0.11-0.89	**0.030**	0.05 (0.52)	1.05 (0.80)	0.38-2.91	0.921
Parental smoking	−0.18 (0.33)	0.84 (0.95)	0.44-1.59	0.585	−0.20 (0.39)	0.82 (0.72)	0.38-1.77	0.607
Sleep disorders	−0.12 (0.34)	0.84 (0.68)	0.43-1.63	0.600	−0.72 (0.39)	0.49 (0.32)	0.23-1.04	0.063
Headache	−0.42 (0.34)	0.66 (0.59)	0.34-1.29	0.220	−0.97 (0.42)	0.38 (0.44)	0.17-0.87	**0.021**
Abdominal pain	0.22 (0.51)	1.25 (1.43)	0.46-3.39	0.661	−0.49 (0.58)	0.61 (0.96)	0.20-1.93	0.403
Headache and abdominal pain	−0.78 (0.46)	0.46 (0.38)	0.19-1.13	0.089	−1.56 (0.51)	0.21 (0.24)	0.08-0.57	**0.002**

adj = adjusted (all explanatory variables included in model).

BMI = body mass index.

OR = Odds ratio.

unadj = unadjusted (only one explanatory variable included in model).

Numbers in bold indicate statistical significance.

**Table 4:** **Predictors for pain in more than one spinal area (logistic regression analysis: 0=no, 1=yes)**

	**Pain in more than one spinal area (N=40) versus no pain (N=160)**			
	**B (SE)**	**OR:**	**95% CI**	**p**_**adj**_
		**Exp B**_**adj**_ **(Exp B**_**unadj**_ **)**	**Exp B**_**adj**_	
Age	0.28 (0.13)	1.33 (1.33)	1.04-1.70	**0.023**
Gender	0.75 (0.43)	2.11 (2.02)	0.91-4.86	0.080
BMI	-0.02 (0.06)	0.98 (1.06)	0.88-1.11	0.790
Finger Floor Distance	-0.02 (0.02)	0.98 (0.99)	0.94-1.03	0.468
Adams sign	-0.06 (0.53)	0.94 (1.67)	0.34-2.66	0.914
Single leg stance with closed eyes	0.44 (0.51)	1.56 (1.04)	0.58-4.21	0.381
TV/computer activities	0.07 (0.03)	1.07 (1.06)	1.01-1.14	**0.031**
Parental back pain	0.38 (0.62)	1.46 (1.29)	0.43-4.92	0.544
Parental smoking	-0.12 (0.50)	0.89 (1.00)	0.34-2.35	0.811
Sleep disorders	1.24 (0.44)	3.44 (3.43)	1.45-8.13	**0.005**
Headache	0.89 (0.46)	2.44 (2.77)	0.99-5.99	0.053
Abdominal pain	-1.00 (0.91)	0.37 (0.69)	0.06-2.18	0.271
Headache and abdominal pain	1.25 (0.62)	3.50 (3.79)	1.05-11.68	**0.042**

adj = adjusted (all explanatory variables included in model).

BMI = body mass index.

OR = Odds ratio.

unadj = unadjusted (only one explanatory variable included in model).

Numbers in bold indicate statistical significance.

Adolescents with occasional mild pain differed significantly in the parameter age from the healthy adolescents. Additionally, there was an observed tendency that their parents also suffered from back problems (Table 4). Adolescents with moderate or severe occasional back pain reported significantly more often headaches in combination with abdominal pain. The subjects with moderate or severe frequent pain (frequent or daily) within the last month were older than those without pain, were mostly female, complained about headaches and abdominal pain and showed significantly reduced ability to stand with closed eyes on one leg. In addition, there was a tendency that they complained more about sleep problems and isolated headaches when compared to pain-free adolescents (Nagelkerke R^2^ = 0.26) (Table 5).

**Table 5:** **Predictors for mild occasional, moderate/severe occasional and moderate/severe frequent pain (multinomial logistic regression analysis)**

	**Mild occasional pain (N = 38) versus no pain (N = 160)**				**Moderate/severe occasional pain (N = 51) versus no pain (N = 160)**				**Moderate/severe frequent pain (N = 47) versus no pain (N = 160)**			
	**B (SE)**	**OR:**	**95% CI**	**p**_**adj**_	**B (SE)**	**OR:**	**95% CI**	**p**_**adj**_	**B (SE)**	**OR:**	**95% CI**	**p**_**adj**_
		**Exp B**_**adj**_ **(Exp B**_**unadj**_ **)**	**Exp B**_**adj**_			**Exp B**_**adj**_ **(Exp B**_**unadj**_ **)**	**Exp B**_**adj**_			**Exp B**_**adj**_ **(Exp B**_**unadj**_ **)**	**Exp B**_**adj**_	
Age	0.34 (0.13)	1.40 (1.35)	1.10-1.79	**0.007**	0.19 (0.11)	1.21 (1.19)	0.98-1.51	0.082	0.35 (0.12)	1.42 (1.34)	1.13-1.78	**0.003**
Gender	0.06 (0.41)	1.06 (1.06)	0.48-2.35	0.885	−0.06 (0.36)	0.94 (0.71)	0.46-1.91	0.867	−0.81 (0.39)	0.45 (0.39)	0.21-0.96	**0.040**
BMI	0.03 (0.06)	1.03 (1.08)	0.91-1.15	0.673	0.07 (0.06)	1.07 (1.09)	0.96-1.19	0.235	−0.01 (0.06)	0.99 (1.08)	0.88-1.11	0.832
FFD	−0.11 (0.02)	0.99 (0.99)	0.95-1.03	0.568	−0.02 (0.02)	0.98 (1.00)	0.95-1.02	0.324	0.001 (0.02)	1.00 (1.00)	0.97-1.04	0.939
Adams sign	−0.70 (0.45)	0.50 (0.44)	0.20-1.20	0.119	0.67 (0.55)	1.96 (1.13)	0.67-5.74	0.219	0.17 (0.49)	1.19 (0.57)	0.46-3.09	0.727
SLS EC	0.33 (0.55)	1.39 (1.07)	0.47-4.12	0.552	−0.07 (0.45)	0.94 (1.42)	0.39-2.28	0.885	−0.90 (0.43)	0.41 (0.74)	0.18-0.94	**0.035**
TV/computer	0.03 (0.03)	1.03 (1.06)	0.98-1.09	0.246	0.03 (0.03)	1.03 (1.03)	0.98-1.51	0.299	0.03 (0.03)	1.03 (1.04)	0.98-1.09	0.261
Parental back pain	−1.38 (0.79)	0.25 (0.32)	0.05-1.19	0.082	−1.01 (0.67)	0.36 (0.34)	0.10-1.34	0.128	0.18 (0.53)	1.20 (0.92)	0.43-3.35	0.732
Parental smoking	0.08 (0.48)	1.09 (1.14)	0.43-2.76	0.859	−0.41 (0.38)	0.66 (0.80)	0.31-1.40	0.283	−0.32 (0.41)	0.73 (0.72)	0.33-1.61	0.434
Sleep disorders	0.30 (0.53)	1.35 (1.00)	0.48-3.77	0.569	−0.40 (0.39)	0.67 (0.55)	0.31-1.45	0.311	−0.74 (0.40)	0.48 (0.32)	0.22-1.05	0.065
Headache	−0.04 (0.47)	0.96 (0.78)	0.39-2.41	0.938	−0.63 (0.41)	0.53 (0.50)	0.24-1.20	0.127	−0.80 (0.44)	0.45 (0.49)	0.19-1.05	0.065
Abdominal pain	0.49 (0.70)	1.64 (1.87)	0.41-6.49	0.483	−0.04 (0.63)	0.97 (1.18)	0.28-3.35	0.956	−0.51 (0.59)	0.60 (0.84)	0.19-1.92	0.389
Head/Abdom pain	1.07 (1.08)	2.93 (3.39)	0.35-24.18	0.319	−1.39 (0.50)	0.25 (0.21)	0.09-0.66	**0.005**	−1.30 (0.55)	0.27 (0.27)	0.09-0.79	**0.017**

adj = adjusted (all explanatory variables included in model).

BMI = body mass index.

FFD = Finger floor distance.

Head/Abdom pain = Headache and abdominal pain.

OR = Odds ratio.

SLS EC = Single leg stance with eyes closed.

unadj = unadjusted (only one explanatory variable included in model).

Numbers in bold indicate statistical significance.

The adolescents who reported spinal pain in more than one spinal area that was of moderate or severe intensity and of high frequency (frequent or daily), were older, mostly female, complained about sleep disorders and showed less ability to stand on one leg with closed eyes than adolescents without pain in the previous month (Nagelkerke R^2^ = 0.34) (Table 6).

**Table 6:** **Predictors for moderate/severe and frequent/daily pain in more than one spinal area (logistic regression analysis: 0=no, 1=yes)**

	**Moderate/severe and frequent/daily pain in more than one spinal area (N=22) versus no pain (N=160)**			
	**B (SE)**	**OR:**	**95% CI**	**p**_**adj**_
		**Exp B**_**adj**_ **(Exp B**_**unadj**_ **)**	**Exp B**_**adj**_	
Age	0.34 (0.16)	1.40 (1.48)	1.02-1.92	**0.039**
Gender	1.42 (0.61)	4.15 (3.65)	1.26-13.67	**0.019**
BMI	-0.06 (0.08)	0.94 (1.06)	0.80-1.11	0.478
Finger Floor Distance	-0.01 (0.03)	0.99 (0.99)	0.94-1.06	0.815
Adams sign	-0.16 (0.69)	0.85 (2.02)	0.22-3.27	0.812
Single leg stance with closed eyes	1.29 (0.65)	3.65 (1.75)	1.03-12.97	**0.046**
TV/computer activities	0.06 (0.04)	1.06 (1.05)	0.98-1.16	0.162
Parental back pain	-0.24 (0.75)	0.79 (0.87)	0.18-3.43	0.752
Parental smoking	0.09 (0.65)	1.10 (1.08)	0.31-3.95	0.886
Sleep disorders	1.57 (0.58)	4.79 (4.04)	1.55-14.80	**0.006**
Headache	1.09 (0.66)	2.98 (2.71)	0.82-10.88	0.098
Abdominal pain	-0.28 (1.04)	0.75 (0.95)	0.10-5.79	0.786
Headache and abdominal pain	1.39 (0.85)	4.00 (4.50)	0.76-21.04	0.102

adj = adjusted (all explanatory variables included in model).

BMI = body mass index.

OR = Odds ratio.

unadj = unadjusted (only one explanatory variable included in model).

Numbers in bold indicate statistical significance.

## Discussion

The first aim of this survey was to provide data on intensity, frequency and localization of adolescent spinal pain as well as its consequences on daily life [9]. Pain intensity was reported on average as moderate, which is in line with former studies using the VAS for pain quantification [14,25], but higher than in a study that assessed pain intensity by six faces as suggested in the Young Spine Questionnaire [9] and rescaled it to a 0–10 scale [26]. Which of these two approaches to assess pain intensity in adolescents is preferable however needs further investigation as it might be dependent on age. Nevertheless, all these studies consistently found a linear relation between pain frequency and pain intensity [14,25,26]. In the present study, about one quarter of those adolescents with back pain indicated frequent pain of moderate or severe intensity, which is slightly more than in the study by Aartun et al. [26]. Although these results are not directly comparable due to methodological differences in the assessment of pain intensity (VAS versus faces) and pain frequency (one month versus one week recall period), these results indicate that about every fourth or fifth adolescent back problem might be serious. Additionally, the present study revealed that 13% of all participants, including the pain-free adolescents, suffered from pain in more than one spinal area. These adolescents also reported significantly higher pain intensity and pain frequency and undertook more changes in daily life than those who complained about pain in only one spinal area. These results indicate that adolescents who report pain in multiple areas should receive special attention as they may be at risk of developing a serious back problem in adulthood. This is supported by the findings of a review by Roth-Isigkeit [3] and is further supported by the finding of this study that sleep disorders are one of the predictors for pain in multiple spinal areas. Sleep disorders in childhood occur together with several psychiatric disorders, predict anxiety/depression disorders later in life [27] and are a feature of widespread pain [8]. The adolescents with pain in more than one spinal area might thus correspond to the ‘LBP and depression/anxiety disorders cluster’ by Beales et al. [8], which was characterized by increased probability for headaches and sleep disorders in addition to LBP. In the present study, sleep disorders co-existed, in tendency, with moderate or severe frequent pain. They were also a strong predictor for those adolescents who complained about frequent and at least moderate pain in more than one spinal area. In contrast, headache, particularly in combination with abdominal pain, emerged from this study as the main predictor for experiencing intense pain, while sleep disorders did not predict pain intensity. Similarly, moderate or severe, but occasional pain co-existed with headaches and abdominal pain, but not with sleep disorders. Thus, adolescents with intense, but only occasional pain might represent the ‘spinal cluster’ by Beales et al. [8], which was featured by a high probability of LBP and increased probability of headaches, but absence of sleep disorders. Although these clusters were defined for LBP only and their significance needs to be followed into adulthood, it might be hypothesized that adolescents of the ‘LBP and depression/anxiety disorders cluster’, in whom back pain represents a multidimensional health problem, should be carefully monitored.

Regarding pain frequency, parental back pain was, apart from age, the only significant risk factor for occasional back pain regardless of intensity and there was a tendency for it to co-exist with mild occasional back pain. Therefore the adolescents with mild occasional back pain might be regarded as healthy [7] and correspond to the ‘healthy individuals cluster’ in the study by Beales et al. [8]. While several studies reported spinal pain in at least one parent to be a risk factor for adolescent spinal pain [14,28], this study revealed that this applies only for the appearance of occasional, but not of frequent back pain. Thus, the impact of the parental role model seems to be of less importance than previously suggested. Frequent spinal pain was predicted, similarly to pain in more than one spinal area, by headache (particularly combined with abdominal pain) and with a tendency for sleep disorders. Interestingly, impaired single leg stance with closed eyes was a further predictor for frequent back pain. This parameter also emerged as the only physical parameter which distinguished pain-free adolescents from those with the more serious back problem (frequent and at least moderate pain in more than one spinal area). Reduced postural stability, predominantly with visual obstruction, has been shown in adults with LBP [21] and neck pain [29], regardless of pain duration. Reduced proprioceptive input due to neurological adaptations or acute pain inhibition was proposed as a possible explanation for this phenomenon [21]. About the causative factors for the observed deficiency in single leg stance in the present study can only be speculated. Neurological adaptations due to chronic pain are less probable in adolescents, and acute pain (point prevalence) was not asked for. Reduced balance control, mainly in visual and somatosensory conflict situations, was reported in adolescent with idiopathic scoliosis at the disease onset [30]. Alternatively, the observed impairment in single leg stance could be an indicator of a general coordinative clumsiness. Regardless of etiology, the importance of balance deficits under visual restriction for the development of back problems in adolescence needs further investigation. Furthermore, the reliability of the chosen balance test needs to be determined in adolescents and the center of pressure sway should be measured in those adolescents with severe and frequent pain in more than one spinal area.

Regardless of pain localization, pain intensity increased in parallel with pain frequency and was the only predictor of whether the experience of spinal pain had an impact on the adolescents’ daily life. This corroborates the finding that for adolescent pain in general, pain intensity is the most robust predictor for functional impairment [31]. Furthermore, changes in daily life might indeed be proxy measures for pain intensity [9]. However, whether they have, in combination with a measure for pain intensity, the potential to distinguish between trivial and significant pain as suggested in literature [9], remains debatable. Based on the results of this study, data on pain intensity need to be complemented by data on pain frequency and pain localization in order to give a comprehensive picture of the severity of an adolescent back problem. Milanese and Grimmer identified in their review three categories of LBP in literature, namely general LBP (no characterization of LBP), chronic/recurrent LBP (characterized by a measure of recurrence) and severe/disabling LBP (characterized by a measure of severity), that might have different risk factors [32]. The results of this study suggest that these categories also need to be studied in combination. In terms of the description of adolescent spinal pain in general, particularly pain localization in more than one spinal area should be recorded.

### Limitations

The main limitation of the present study was that it used a non-validated questionnaire. The ‘Young Spine Questionnaire’ (YSQ), which has meanwhile been developed [9], has so far been tested on children in the age of 9 to 11 years and is available only in English and Danish. Therefore, it was not chosen for this study. However, the composition of the questionnaire used in this study was similar in terms of dividing the three spinal regions neck, mid back and lower back and illustrating them by means of a manikin. The main differences were that the YSQ asked for the 1-week period prevalence, whereas the present study used the 1-month period prevalence as recommended by other studies [7,11]. Nevertheless, to compare results, the 1-week period prevalence may be used in the future. Furthermore, similar to the YSQ, the questionnaire should include a question on point prevalence (pain today). A second major limitation was that the participants were volunteers, which implies a selection bias. Therefore, the results of the study may not be generalized to the whole Swiss adolescent population. Furthermore, although the total number of participants was high, the sample size of some categories became rather small, which might have increased the risk for not detecting some associations (type II error).

## Conclusions

Studies on adolescent spinal pain should report data on pain frequency, intensity and localization. While the exclusive information on pain intensity seems to be of less importance, all adolescents who present with pain in more than one spinal area or report frequent pain should be followed carefully. Reduced balance with visual deprivation (closed eyes) might be a physical indicator of a serious back problem. To determine its relevance, however, this parameter needs being followed in longitudinal studies.

## Additional file

Additional file 1:
**Questionnaire.**

## References

[1] Jeffries LJ, Milanese SF, Grimmer-Somers KA (2007). Epidemiology of adolescent spinal pain: a systematic overview of the research literature. Spine.

[2] Kjaer P, Wedderkopp N, Korsholm L, Leboeuf-Yde C (2011). Prevalence and tracking of back pain from childhood to adolescence. BMC Musculoskelet Disord.

[3] Roth-Isigkeit A, Schwarzenberger J, Baumeier W, Meier T, Lindig M, Schmucker P (2005). Risk factors for back pain in children and adolescents. Schmerz.

[4] Leboeuf C (1998). At what age does low back pain become a problem?. Spine.

[5] Hestbaek L, Leboeuf-Yde C, Kyvik KO, Manniche C (2006). The course of low back pain from adolescence to adulthood: eight-year follow-up of 9600 twins. Spine.

[6] Cardon G, Balague F (2004). Low back pain prevention’s effects in schoolchildren. What is the evidence?. Eur Spine J.

[7] Pellise F, Balague F, Rajmil L, Cedraschi C, Aguirre M, Fontecha CG (2009). Prevalence of low back pain and its effect on health-related quality of life in adolescents. Arch Pediatr Adolesc Med.

[8] Beales DJ, Smith AJ, O’Sullivan PB, Straker LM (2012). Low back pain and comorbidity clusters at 17 years of age: a cross-sectional examination of health-related quality of life and specific low back pain impacts. J Adolesc Health.

[9] Lauridsen HH, Hestbaek L (2013). Development of the young spine questionnaire. BMC Musculoskelet Disord.

[10] Watson KD, Papageorgiou AC, Jones GT, Taylor S, Symmons DP, Silman AJ (2003). Low back pain in schoolchildren: the role of mechanical and psychosocial factors. Arch Dis Child.

[11] Watson KD, Papageorgiou AC, Jones GT, Taylor S, Symmons DP, Silman AJ (2002). Low back pain in schoolchildren: occurrence and characteristics. Pain.

[12] Balague F, Troussier B, Salminen JJ (1999). Non-specific low back pain in children and adolescents: risk factors. Eur Spine J.

[13] Feldman DE, Rossignol M, Shrier I, Abenhaim L (1999). Smoking. A risk factor for development of low back pain in adolescents. Spine.

[14] Wirth B, Knecht C, Humphreys K (2013). Spine Day 2012: spinal pain in Swiss school children- epidemiology and risk factors. BMC Pediatr.

[15] Jones GT, Watson KD, Silman AJ, Symmons DP, Macfarlane GJ (2003). Predictors of low back pain in British schoolchildren: a population-based prospective cohort study. Pediatrics.

[16] Lee CF, Fong DY, Cheung KM, Cheng JC, Ng BK, Lam TP (2010). Referral criteria for school scoliosis screening: assessment and recommendations based on a large longitudinally followed cohort. Spine.

[17] Kovacs FM, Gestoso M, del Gil Real MT, Lopez J, Mufraggi N, Mendez JI (2003). Risk factors for non-specific low back pain in schoolchildren and their parents: a population based study. Pain.

[18] Nissinen M, Heliovaara M, Seitsamo J, Alaranta H, Poussa M (1994). Anthropometric measurements and the incidence of low back pain in a cohort of pubertal children. Spine.

[19] Hill J, Keating J (2010). Risk factors for the first episode of low back pain in children are infrequently validated across samples and conditions: a systematic review. J Physiother.

[20] Wyss T, Marti B, Rossi S, Kohler U, Mäder U (2007). Assembling and verification of a fitness test battery for the recruitement of the Swiss army and nation-wide use. Schweizerische Zeitschrift für Sportmedizin und Sporttraumatologie.

[21] Ruhe A, Fejer R, Walker B (2011). Center of pressure excursion as a measure of balance performance in patients with non-specific low back pain compared to healthy controls: a systematic review of the literature. Eur Spine J.

[22] Harringe ML, Halvorsen K, Renstrom P, Werner S (2008). Postural control measured as the center of pressure excursion in young female gymnasts with low back pain or lower extremity injury. Gait Posture.

[23] Perret C, Poiraudeau S, Fermanian J, Colau MM, Benhamou MA, Revel M (2001). Validity, reliability, and responsiveness of the fingertip-to-floor test. Arch Phys Med Rehabil.

[24] Hirschfeld G, Zernikow B (2013). Cut points for mild, moderate, and severe pain on the VAS for children and adolescents: what can be learned from 10 million ANOVAs?. Pain.

[25] Stahl M, Mikkelsson M, Kautiainen H, Hakkinen A, Ylinen J, Salminen JJ (2004). Neck pain in adolescence. A 4-year follow-up of pain-free preadolescents. Pain.

[26] Aartun E, Hartvigsen J, Wedderkopp N, Hestbaek L (2014). Spinal pain in adolescents: prevalence, incidence, and course: a school-based two-year prospective cohort study in 1,300 Danes aged 11–13. BMC Musculoskelet Disord.

[27] Shanahan L, Copeland WE, Angold A, Bondy CL, Costello EJ (2014). Sleep problems predict and are predicted by generalized anxiety/depression and oppositional defiant disorder. J Am Acad Child Adolesc Psychiatry.

[28] Kaspiris A, Grivas TB, Zafiropoulou C, Vasiliadis E, Tsadira O (2010). Nonspecific low back pain during childhood: a retrospective epidemiological study of risk factors. J Clin Rheumatol.

[29] Ruhe A, Fejer R, Walker B (2011). Altered postural sway in patients suffering from non-specific neck pain and whiplash associated disorder - A systematic review of the literature. Chiropr Man Therap.

[30] Haumont T, Gauchard GC, Lascombes P, Perrin PP (2011). Postural instability in early-stage idiopathic scoliosis in adolescent girls. Spine.

[31] Roth-Isigkeit A, Thyen U, Stoven H, Schwarzenberger J, Schmucker P (2005). Pain among children and adolescents: restrictions in daily living and triggering factors. Pediatrics.

[32] Milanese S, Grimmer-Somers K (2010). What is adolescent low back pain? Current definitions used to define the adolescent with low back pain. J Pain Res.

