# Supplementary material for: Pain characteristics of adolescent spinal pain
Source: BMC Pediatr. 2015 Apr 17;15:42. doi: 10.1186/s12887-015-0344-5 (PMC4411747; doi:10.1186/s12887-015-0344-5)
Supplement: Additional file 1: — Questionnaire. [file 12887_2015_344_MOESM1_ESM.doc]

Additional file 1: Questionnaire

1. Did you ever have pain in your back or neck?
2. Did you have pain in your back or neck in the last four weeks?
3. If you experienced pain in the last four weeks: How often did you have pain?
4. If you experienced pain in the last four weeks: How severe was your pain (VAS)?
5. If you experienced pain in the last four weeks: Was the pain so strong that you had to change something in your daily life?

If so, what did you change: reduction of leisure/sport activities, school absence, seeing a doctor/ chiropractor, taking medication, other measures (what).

1. Do your parents suffer from back or neck pain?
2. Do you smoke or do your parents smoke?
3. How many hours per week do you sit in leisure time in front of the computer or the TV?
4. Did you have sleep disorders (falling asleep or sleep through the night) in the last four weeks?
5. Did you have headaches or abdominal pain (apart from period pains) in the last four weeks?
